# Disulfide-crosslink scanning reveals prion–induced conformational changes and prion strain–specific structures of the pathological prion protein PrP^Sc^

**DOI:** 10.1074/jbc.RA117.001633

**Published:** 2018-06-22

**Authors:** Yuzuru Taguchi, Li Lu, Cristobal Marrero-Winkens, Hiroki Otaki, Noriyuki Nishida, Hermann M. Schatzl

**Affiliations:** From the ‡ Department of Comparative Biology and Experimental Medicine, Faculty of Veterinary Medicine, University of Calgary, Calgary, Alberta T2N 4Z6, Canada,; the § Department of Molecular Microbiology and Immunology, Division of Cellular and Molecular Biology, Graduate School of Biomedical Sciences, Nagasaki University, Nagasaki 852-8523, Japan,; the ¶ Calgary Prion Research Unit, University of Calgary, Calgary, Alberta, Canada, and; the ‖ Center for Bioinformatics and Molecular Medicine, Graduate School of Biomedical Sciences, Nagasaki University, Nagasaki 852-8523, Japan

**Keywords:** prion, prion disease, protein crosslinking, protein misfolding, protein structure, protein conformation, prion conversion, prion protein

## Abstract

Prions are composed solely of the pathological isoform (PrP^Sc^) of the normal cellular prion protein (PrP^C^). Identification of different PrP^Sc^ structures is crucially important for understanding prion biology because the pathogenic properties of prions are hypothesized to be encoded in the structures of PrP^Sc^. However, these structures remain yet to be identified, because of the incompatibility of PrP^Sc^ with conventional high-resolution structural analysis methods. Previously, we reported that the region between the first and the second α-helix (H1∼H2) of PrP^C^ might cooperate with the more C-terminal side region for efficient interactions with PrP^Sc^. From this starting point, we created a series of PrP variants with two cysteine substitutions (C;C-PrP) forming a disulfide-crosslink between H1∼H2 and the distal region of the third helix (Ctrm). We then assessed the conversion capabilities of the C;C-PrP variants in N2a cells infected with mouse-adapted scrapie prions (22L-ScN2a). Specifically, Cys substitutions at residues 165, 166, or 168 in H1∼H2 were combined with cysteine scanning along Ctrm residues 220–229. We found that C;C-PrPs are expressed normally with glycosylation patterns and subcellular localization similar to WT PrP, albeit differing in expression levels. Interestingly, some C;C-PrPs converted to protease-resistant isoforms in the 22L-ScN2a cells, but not in Fukuoka1 prion-infected cells. Crosslink patterns of convertible C;C-PrPs indicated a positional change of H1∼H2 toward Ctrm in PrP^Sc^–induced conformational conversion. Given the properties of the C;C-PrPs reported here, we propose that these PrP variants may be useful tools for investigating prion strain–specific structures and structure–phenotype relationships of PrP^Sc^.

## Introduction

Prions are unconventional pathogens composed solely of aberrantly-folded isoforms (PrP^Sc^)^3^ of cellular prion protein (PrP^C^) devoid of any nucleotide genome. Prions cause fatal neurodegenerative disorders in various mammalian species, *e.g.* Creutzfeldt-Jakob disease (CJD) in humans, scrapie in sheep and goat, chronic wasting disease (CWD) in cervids, and bovine spongiform encephalopathy in cattle (1). Despite the lack of a nucleotide genome, prions behave like viruses in terms of *quasi-species* nature, high specificity of host ranges, and diversity in clinicopathological features that are stably inherited over generations (2). Their pathogenic traits are thought to be enciphered in the structures of PrP^Sc^ (3) and high-fidelity representation of the structures on the nascent PrP^Sc^ through a template-guided refolding of PrP^C^ by the template PrP^Sc^ enables faithful inheritance of the traits. Elucidation of details of the structures of PrP^Sc^ and the refolding process are therefore essential for prion research, but they are yet to be decoded because PrP^Sc^ is unsuitable for conventional high-resolution structural analyses. Alternatively, structural models of PrP^Sc^ were deduced based on secondary-structural information of PrP^Sc^ obtained with FTIR spectroscopy, hydrogen/deuterium exchange analysis (4, 5), or images of EM on two-dimensional crystals or fibrils of purified PrP^Sc^ (6–8). Structural differences between prion strains were also inferable from varied biochemical properties of PrP^Sc^, *e.g.* molecular size of proteinase K (PK)-resistant fragments (PK-res) (9), structural stabilities in denaturant solutions (10, 11), and glycoform ratios (12). As another approach, Hafner-Bratkovic and colleagues utilized disulfide-crosslinking of recombinant PrPs to identify regions that maintain structures throughout the aggregation formation process (13).

**Figure 1.**
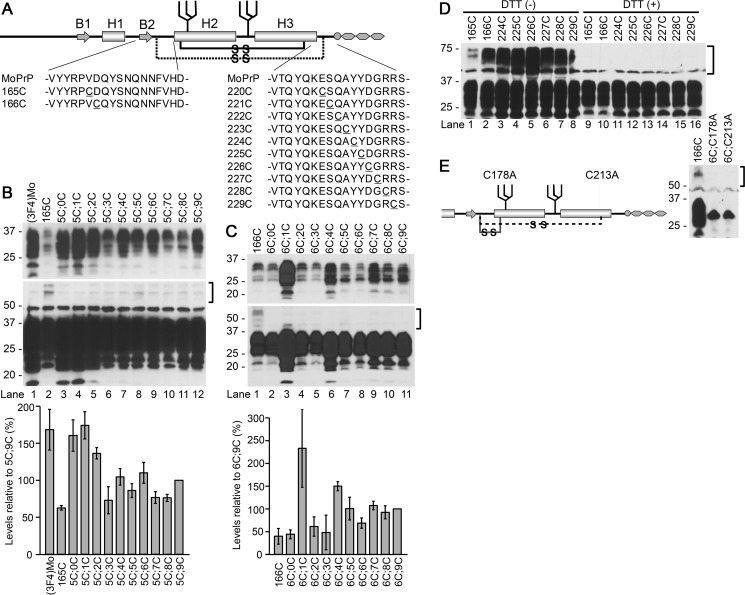
**Design and expression of mutant PrPs with two cysteine (Cys) substitutions, 165C;C- and 166C;C-series.** *A,* a schematic illustration of the secondary-structure components of mouse PrP and positions of the substituted cysteines (Cys). *MoPrP*, sequences of WT mouse PrP. *B1* and *B2*, the first and the second β strands, respectively. *H1*, *H2,* and *H3*, the first, second, and third α helices (46). 165C or 166C was combined with another Cys scanning the distal H3 (Ctrm) from residue 220 to 229 (165C;C- or 166C;C-series, respectively; substituted Cys are *underlined*). The *solid* and *broken lines* with -S-S- represent the native and the newly introduced disulfide-crosslink, respectively. *B* and *C,* expression levels and PrP-banding patterns of the 165C;C- and 166C;C-series. Immunoblots were developed with anti-PrP mAb 3F4 from whole-cell lysates of transiently transfected N2a cells. Each two-Cys construct is named after the last digits of the residue numbers of the substituted Cys (in mouse numbering). 165C or 166C, mutants with Cys substitution only at position 165 or 166, respectively. *Square brackets* indicate positions of dimeric forms of the mutant PrPs. The *upper* and *lower panels* of blots represent images of short- and long-exposure of the same PVDF membranes, respectively. Note that all constructs have similar banding patterns as (3F4)MoPrP, indicating complex-type *N*-linked glycosylation. Two-Cys mutants show trace amounts of dimmers, whereas 165C and 166C have discernible dimers despite their low expression levels. *Bottom panels* are graphs showing expression levels of each series quantified by densitometry. Each *bar* represents mean ± S.D. from three independent experiments. *D,* single-Cys PrPs have substantial levels of PrP dimer formation that disappear after DTT treatment. Immunoblots were probed with 3F4 mAb showing banding patterns of mutant PrPs with only a single Cys substitution either in H1∼H2 or Ctrm. *DTT* (+) and (−) samples, with or without DTT in the sample buffer. The *square bracket* indicates the position of the dimeric forms. *E,* disruption of the native disulfide bond drastically changes the banding pattern, suggesting that the substituted Cys in the 165C;C- and 166C;C-series do not affect the native disulfide bond formation. *Left panel*, scheme illustrating the positions of alanine substitutions for the native Cys. The *solid* or *broken lines* with -S-S- represent putative disulfide-crosslinks of 6C;C213A or 6C;C178A, respectively. *Right panel*, immunoblots with 3F4 mAb showing PrP banding patterns of the mutant PrPs combining 166C with the alanine substitution. Note that the banding patterns of 6C;C178A and 6C;C213A are very different from that of 166C, presumably due to high-mannose-type *N*-glycosylation, unlike the banding patterns of 165C;C- or 166C;C-series constructs.

Unlike PrP^Sc^, high water-solubility and small molecular size of PrP^C^ allowed detailed structural analysis by NMR spectroscopy. The global three-dimensional structures of PrP^C^ are highly conserved among different species with the same secondary-structure components, *i.e.* two short β strands (Fig. 1, *A, B1*, and *B2*) and three α helices (H1, H2, and H3) (14–16). Interspecies variations in amino acid sequences tend to cluster at some spots including the region between H1 and H2 (H1∼H2) or near the C-terminal glycosylphosphatidylinositol anchor-attachment site (Ctrm) (17), and affect interspecies transmission of prions (18, 19). For example, asparagine at the codon 170 can greatly affect interspecies transmissions of prions; transmission of CWD to transgenic mice expressing an elk/mouse chimeric PrP with mouse residues only in Ctrm was substantially inefficient (20). Moreover, a polymorphism in Ctrm of cervid PrP influences stability of CWD strains (21).

We previously demonstrated that efficiencies of dominant-negative inhibition by mutant PrPs with internal deletions in the H1∼H2 region (ΔPrP) correlated with the deletion sizes, proposing that this region might be an interaction interface for PrP^C^–PrP^Sc^ interactions (22). Positional relationships between H1∼H2 and Ctrm seemed important for ΔPrPs to efficiently interact with PrP^Sc^. Inspired by those findings, we hypothesized that the positional relationship of H1∼H2 with Ctrm influences PrP^C^–PrP^Sc^ interactions and subsequent prion conversion. To test this hypothesis, we created a series of mutant PrPs with two cysteine (Cys) substitutions (C;C-PrP), one in H1∼H2 and the other in Ctrm. This crosslinks the two regions by an artificial disulfide bond. Effects of these additional bonds on PrP^C^–PrP^Sc^ conversion were monitored in prion-infected cells. Disulfide-crosslink is particularly useful in combination with Cys scanning, systematically tying up the two regions in various fashions. Those intramolecularly crosslinked PrPs were normally expressed on the cell surface when expressed in N2a cells persistently infected with mouse-adapted scrapie (22L-ScN2a), some of them converted into PK-resistant isoforms in a PrP^Sc^-dependent manner. Interestingly, convertibility of the mutants crucially depended on certain patterns of crosslinks. Furthermore, the convertibility of C;C-PrP seemed to be strain-dependent, suggesting that this region is involved in creating prion-strain diversity. Our unique approach provides novel insights into the structural requirements for PrP^c^–PrP^Sc^ conversion.

## Results

### Design of C;C-PrP series

To assess the significance of the intramolecular interactions between H1∼H2 and Ctrm on PrP^C^–PrP^Sc^ interactions and the subsequent conversion, we created a series of mutant PrPs that have two Cys substitutions, one in H1∼H2 and the other in Ctrm so that the two regions are crosslinked by an artificial disulfide bond (Fig. 1*A*), and tested their conversion to PK-res forms. For the Cys substitution in H1∼H2, we selected Val-165 and Asp-166 (residues were numbered according to mouse numbering unless otherwise noted), because they are close enough to Ctrm to form a stable disulfide-crosslink in a native PrP^C^ conformation (PDB code 2L39) (14). Indeed, the global conformation of a mutant human PrP with an extra disulfide bond between residues 166 and 221 (in human numbering; they are equivalent to 165 and 220 of mouse PrP, respectively) were similar to that of WT human PrP (25, 26). The second Cys substitution scanned Ctrm from residue 220 to 229. C;C-PrP constructs are named after the positions of Cys but only the last-digit numbers were used for simplicity, *e.g.* a mutant with Cys at 165 and 229 is named as “5C;9C.” Because a 3F4 epitope-tagged mouse PrP ((3F4)MoPrP) was used as the template for site-directed mutagenesis, every mutant PrP carries a 3F4 epitope tag.

To assess possible structural consequences of mutations and to estimate influences of the disulfide-crosslinks on PrP structure, we analyzed representative C;C-PrPs by short molecular dynamic simulations (Fig. S1). Interestingly, the global structures of C;C-PrPs were not severely distorted by the crosslinks.

### Expression levels, glycosylation, and subcellular localization of C;C-PrP series

We transfected N2a mouse neuroblastoma cells with plasmids encoding the 165C;C- and 166C;C-series (Fig. 1*A*) to examine expression levels and glycosylation status of the mutant PrPs. Banding patterns of all mutants were similar to that of (3F4)MoPrP (Fig. 1*B*), typical of PrP^C^ with complex-type *N*-linked glycans and glycosylphosphatidylinositol anchor, and lacked the dimeric forms (Fig. 1*B*, *square bracket*). Their expression levels were varied (Fig. 1*B*, *graphs*). A C;C-PrP that has Cys residues at positions equivalent to those of the aforementioned human PrP mutant (25), namely 5C;0C, showed the highest expression level comparable with (3F4)MoPrP (Fig. 1*B*, *lane 2*), presumably because its intramolecular disulfide-crosslink between the substituted Cys did not interfere with the native PrP^C^ conformation. “Intramolecular” disulfide-crosslink of C;C-PrPs is implied by the absence of discernible dimeric forms. The mutant PrPs with a single Cys substitution formed substantial levels of dimeric forms that are presumably crosslinked by a intermolecular disulfide bond and disappear upon dithiothreitol (DTT) treatment (Fig. 1*D*, DTT(−) *versus* (+)). The 166C;C-series mutants showed similar banding patterns as 165C;C-series without discernible dimeric forms (Fig. 1*C*, *square bracket*). 6C;1C and 6C;4C showed the highest expression levels among the 166C;C-series (Fig. 1*C*, *lanes 3* and *6*).

**Figure 2.**
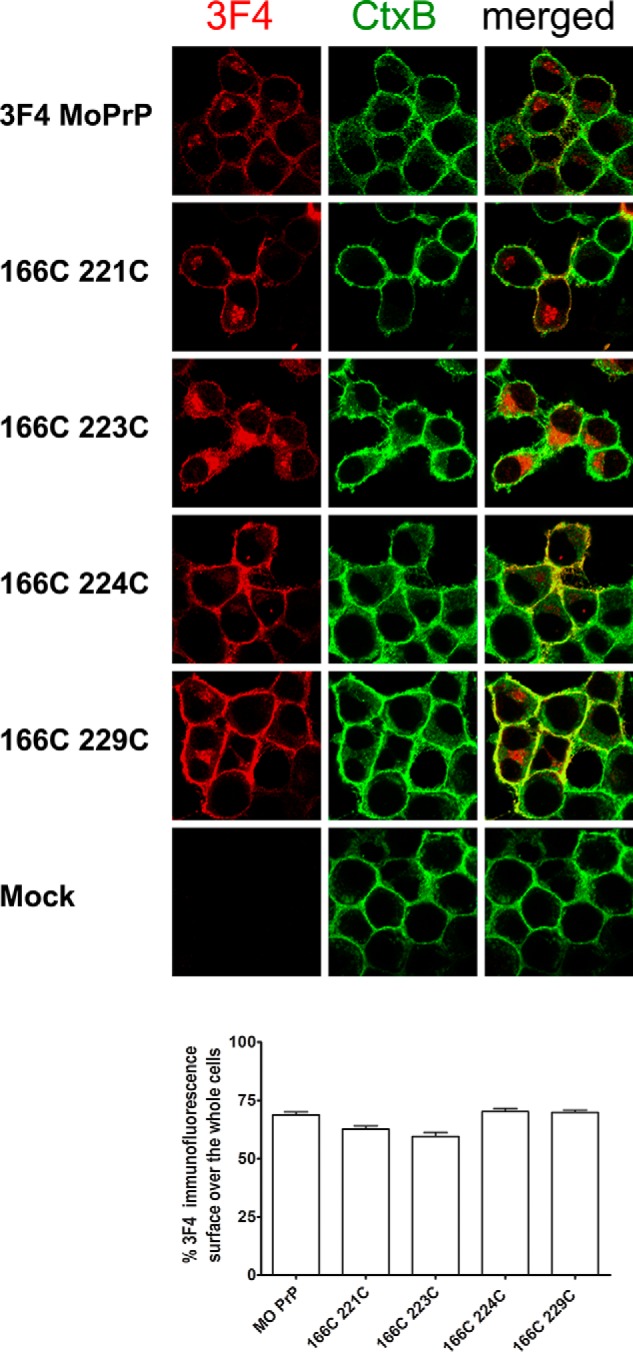
**Confocal microscopy analysis of relative surface expression levels of representative 166C;C-PrPs correlated to CtxB surface staining.** CtxB live cell-stained transfected cells were fixed and permeabilized, and then analyzed for 3F4-PrP expression. 3F4-PrP expression is shown in *red*, CtxB staining in *green*, and the *right panels* show merged signals. The *upper panels* show (3F4)MoPrP-transfected cells as positive control, and *lower panels* show mock-transfected cells as negative control. Overlaying the CtxB signals with 3F4-PrP expression was used to quantify the relative surface expression levels of C;C-PrPs (*lower graph*). Constructs were detected at similar rates at the cell surface, excluding major differences in subcellular localization.

To rule out the possibility that the Cys residues at 178 and 213, which contribute to the native disulfide bond might be shuffled to couple with the substituted Cys, we replaced either Cys at 178 or 213 with alanine so that the native disulfide bond is broken and instead coupled with 166C (6C;C178A and 6C;C213A) (Fig. 1*E*, *schematic*). The banding patterns of those mutants were very different from that of WT PrP, reminiscent of PrP with high-mannose–type *N*-linked glycans (27) (Fig. 1*E*, *right panel*). Absence of those features supported that C;C-PrPs form an intramolecular disulfide-crosslink between the substituted Cys without affecting the native disulfide and undergo normal folding and processing in ER and trans-Golgi network. In accordance with this view, immunofluorescence analysis demonstrated that C;C-PrPs were distributed on the cell surface (Fig. S2, nonpermeabilized) and in perinuclear regions as clusters (Fig. S2, permeabilized) like WT PrP, corroborating normal intracellular trafficking and subcellular localization of C;C-PrPs. To further exclude aberrant localization of C;C-PrPs, we correlated the expression of transfected C;C-PrPs (mAb 3F4 positive) with cholera toxin B (CtxB) labeling of cell-surface lipid rafts using confocal microscopy (Fig. 2). Overlaying and merging the 3F4 and CtxB signals allowed us to quantify how much transfected C;C-PrP is located at the cell surface or in intracellular compartments. When comparing the percentage rates of surface expression for various C;C-PrP to (3F4)MoPrP, we found for all constructs similar relative surface expression levels (Fig. 2). Taken together, these data indicate that C;C-PrPs undergo normal cellular trafficking and localization within cells.

### Evidence for intramolecular disulfide-crosslink formation

**Figure 3.**
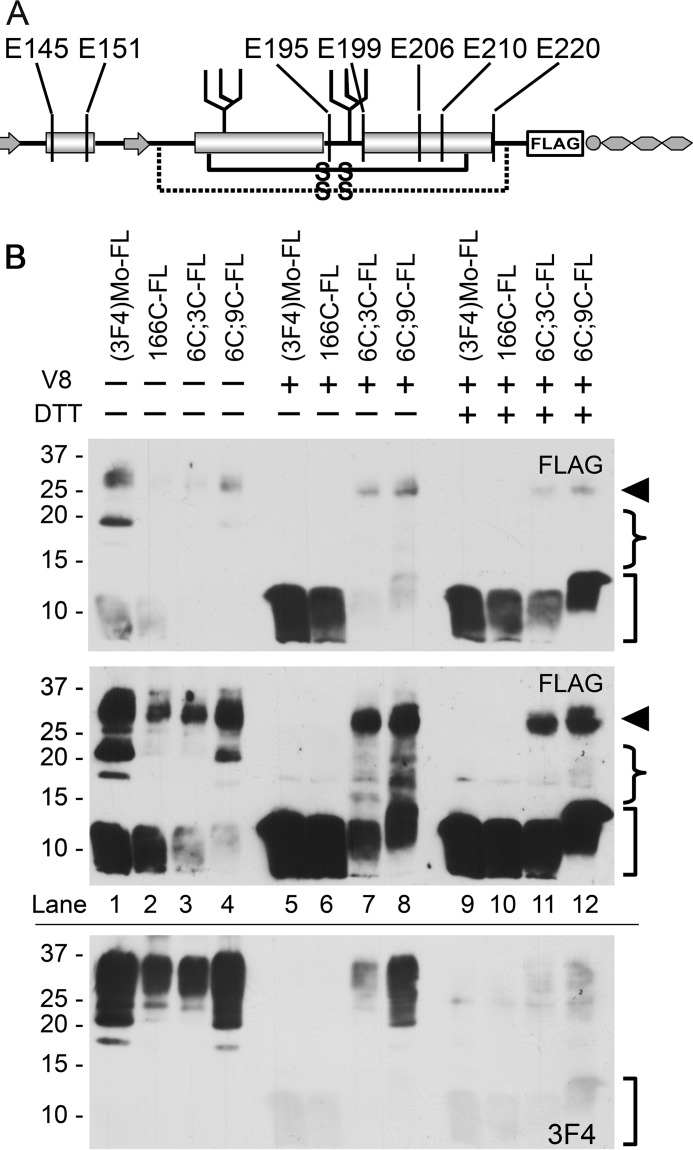
**Substituted Cys of 166C;C-constructs form an intramolecular disulfide-crosslink: assessment of fragment patterns after V8-protease digestion.** *A,* a schematic illustrating the position of the FLAG-tagged 166C;C-constructs along with putative cleavage sites by V8-protease (*vertical lines*). *B,* V8-digested fragment profiles are similar between FLAG-tagged WT ((3F4)Mo-FL) and 166C-FLAG (166C-FL), but those of 6C;3C-FLAG and 6C;9C-FLAG (6C;3C-FL and 6C;9C-FL, respectively) are different. Immunoblots were probed with anti-FLAG polyclonal antibody, or 3F4-mAb, showing nondigested and V8-digested PrPs, with or without DTT in the sample buffer. The *upper* and *middle panel* images were obtained from the same PVDF membrane with shorter and longer exposure, respectively. The *bottom panel* shows immunoblots re-probed with 3F4 mAb. *Arrowhead* indicates full-length FLAG-tagged PrP molecules, *curved brackets* are positions of the intermediate-size fragments that diminish by DTT, and *square brackets* indicate the smallest fragments.

To demonstrate intramolecular disulfide-crosslink formation by the substituted Cys, we introduced a FLAG tag to C;C-PrPs (Fig. 3*A*) and analyzed the fragment patterns of V8 protease-digested products. A crosslink between H1∼H2 and Ctrm theoretically produces extra bands on immunoblots by bonding fragments (Fig. 3*A*). Indeed, V8-digested FLAG-tagged (3F4)MoPrP, 166C, 6C;3C, and 6C;9C (Fig. 3*B*) showed distinct banding patterns along with findings suggestive of intramolecular disulfide-crosslink. First, full-length 6C;3C-FLAG and 6C;9C-FLAG PrP molecules remained after the digestion (Fig. 3*B*, compare *lanes 7* and *8*, *arrowhead*), whereas full-length (3F4)Mo-FLAG and 166C-FLAG PrPs were completely digested (Fig. 3*B*, compare *lanes 5* and *6*, *arrowhead*). Relative protease resistance of C;C-PrPs was also implied by smaller amounts of fragments produced by endogenous proteolysis (Fig. 3*B*, *lanes 3* and *4*, *square bracket*). These are attributable to steric effects caused by the crosslink of H1∼H2 and Ctrm, concealing protease-vulnerable regions. Second, the greatly improved immunoreactivity of the smallest fragments of 6C;3C-FLAG and 6C;9C-FLAG by DTT treatments (Fig. 3*B*, *lanes 7 versus 11* or *8 versus 12*) also indicates steric effects hiding the epitope, and its re-exposure by DTT, which breaks apart the crosslinked fragments. Third, the intermediate size fragments of V8-digested 6C;3C-FLAG and 6C;9C-FLAG (Fig. 3*B*, *arrowhead* and *square bracket*, respectively), which disappeared by DTT (Fig. 3*B*, *lanes 7* and *8*, *curled bracket*) apparently represent the predicted “extra fragments” (Fig. 3*B*, *lanes 5* and *6*, *square bracket*). Taken together, these findings strongly support the intramolecular crosslink formation between the introduced Cys residues.

### Conversion of C;C-PrPs into PK-res isoforms by bona fide PrP^Sc^

**Figure 4.**
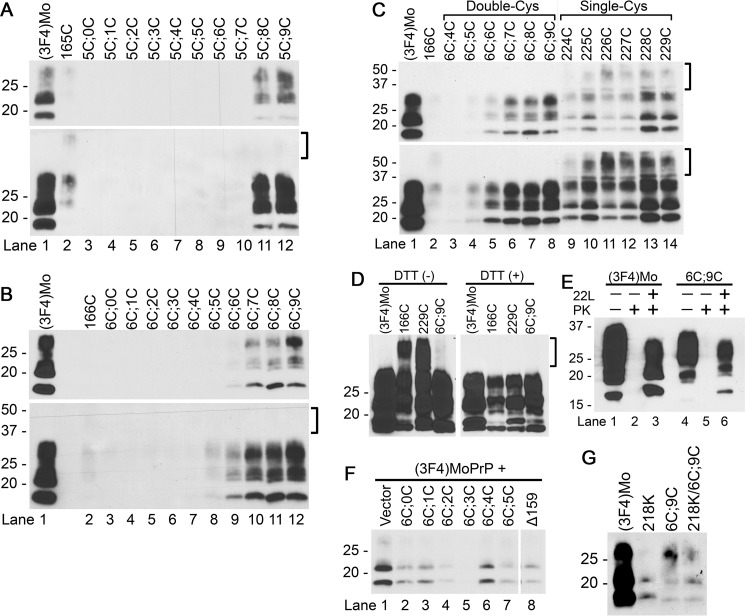
**165C;C- and 166C;C-series mutants convert to PK-resistant PrP forms (PK-res) in 22L-ScN2a cells.** The *upper* and *lower panels* represent images of short- and long-exposure of the same PVDF membranes, respectively. The *square brackets* indicate the position of the dimeric form. *A* and *B,* PK-res PrP^Sc^ of 165C;C- and 166C;C-series, respectively. Immunoblots probed with 3F4 mAb demonstrating the levels of PK-res PrP^Sc^. Just as for PrP^C^ isoforms, PK-res PrP^Sc^ of the C;C-series lacks the dimeric forms. *C,* PK-res PrP of 166C;C-series maintain the intramolecular disulfide-crosslinks throughout conversion. Immunoblots were developed with 3F4 mAb showing PK-res PrP^Sc^ of 166C;C-series and mutant PrPs with a single substituted Cys, either at 166C or in Ctrm. Note that all the single-Cys constructs have substantial levels of dimeric forms, in contrast to 166C;C-series. *D,* the dimeric forms of PK-res PrP^Sc^ of single-Cys mutants disappear after DTT treatment, proving intermolecular disulfide-crosslinks. Immunoblots with 3F4 mAb comparing single-Cys PrP, 166C and 229C, and a double-Cys PrP, 6C;9C. *DTT* (+) and (-) indicate samples prepared with or without DTT in the sample buffer. *E,* PK-res PrP formation of 6C;9C is PrP^Sc^-dependent. Immunoblots with 3F4 mAb demonstrating PK resistance in the lysates from 22L prion-infected and noninfected N2a cells, transiently-transfected with (3F4)MoPrP or C;9C. *22L* (+) or (−) indicates samples from 22L-infected or noninfected N2a cells. *PK* (+) or (−) indicates samples with or without PK digestion. Note that there is no detectable PK-resistant PrP in lysates from noninfected N2a cells. *F,* conversion-incompetent C;C-PrPs can interact with PrP^Sc^. Immunoblots with 3F4 mAb showing efficient dominant-negative inhibition effects on co-transfected (3F4)MoPrP by the conversion-incompetent C;C-PrPs, namely 6C;0–6C;5C. Δ159, a deletion mutant PrP lacking residue 159 as a control (22), was on the same membrane. Note that unnecessary lanes were eliminated. *G,* the conversion reaction of 6C;9C is relatively resistant to the inhibitory effects of the Q218K substitution. Immunoblots with 3F4 mAb comparing PK-res PrP of WT or 6C;9C and their Q218K counterparts. Decrease of PK-res formation by Q218K is smaller in 6C;9C than that in WT.

Next, we assessed conversion efficiencies of 165C;C- and 166C;C-series PrP mutants by expressing them in persistently infected 22L-ScN2a cells and evaluating their PK-resistant forms (PK-res). Among 165C;C-series, only 5C;8C and 5C;9C showed PK-resistant PrP (Fig. 4*A*), whereas the 166C;C-series exhibited gradually increasing levels of PK-res from 6C;5C to 6C;9C (Fig. 4*B*). Just as PrP^C^ isoforms, PK-res of C;C-PrPs lacked dimeric forms (Fig. 4*C*, *double-Cys*), whereas every single-Cys PrPs tested showed intense dimeric bands (224–229C; Fig. 4*C*, *single-Cys*), which disappeared with DTT treatment (Fig. 4*D*). These data support the view that the PK-res molecules of C;C-PrPs were not derived from PrP^C^ isoforms with free Cys residues, *i.e.* without crosslink, but from those with the intramolecular disulfide-crosslink. Absence of PK-res in noninfected N2a cells demonstrated that the conversion of the 6C;9C into PK-res isoform was PrP^Sc^-dependent (Fig. 4*E*, *lane 5*). We hypothesize that the nonconvertible C;C-PrPs, *e.g.* those from 6C;0C to 6C;4C, cannot convert because of their unsuitable positioning of H1∼H2 for efficient refolding as discussed later, but there also was the possibility that they cannot appropriately interact with the PrP^Sc^ template. We tested this possibility by assessing their dominant-negative inhibition efficiencies, as done previously by us for other PrP mutants (22). We had characterized the interaction efficiencies of ΔPrP-series mutants, which have various lengths of internal deletions (from residue 159 to 175) in the H1∼H2 region, with PrP^Sc^ template by evaluating their dominant-negative inhibition efficiencies on co-expressed (3F4)MoPrP (22). Because 6C;0C to 6C;4C would not show discernible PK-res molecules, these constructs could be used without any epitope modification. Not unexpected, 6C;0C to 6C;5C PrPs exhibited as efficient dominant-negative inhibition on co-expressed convertible (3F4)MoPrP as Δ159, which is the most potent inhibitory molecule among the analyzed ΔPrP-series (Fig. 4*F*, *lanes 2–7*), confirming that these C;C-PrPs do interact with template PrP^Sc^ but cannot convert into PK-res isoforms.

### The disulfide-crosslink suppresses the influence of Q218K substitution on PrP^Sc^ conversion

Lysine at codon 219 (in human numbering; Lys-219) is a polymorphism of human PrP well-known for protective effects against sporadic CJD (28). The equivalent substitution in mouse PrP (Q218K) is also protective against mouse-adapted scrapie (29). These effects were explained by the inability of Lys-219 PrP to convert into PrP^Sc^ and its dominant-negative inhibition on the coexisting WT PrP (30). As the effects of Lys-219 were attributed to alteration in structures of the B2-H2 loop (31) (32), we tested whether a mutant PrP combining 6C;9C and Q218K (218K/6C;9C) can convert to PK-res in 22L-ScN2a cells. Interestingly, 218K/6C;9C showed similar PK-res levels as 6C;9C (Fig. 4*G*, *lanes 3 versus 4*), whereas Q218K PrP showed much lower levels compared with WT PrP (Fig. 4*G*, *lanes 1 versus 2*). This suggested that the artificial disulfide-crosslink of 6C;9C can suppress the effects of Q218K.

### Prediction of another C;C-PrP that can convert

**Figure 5.**
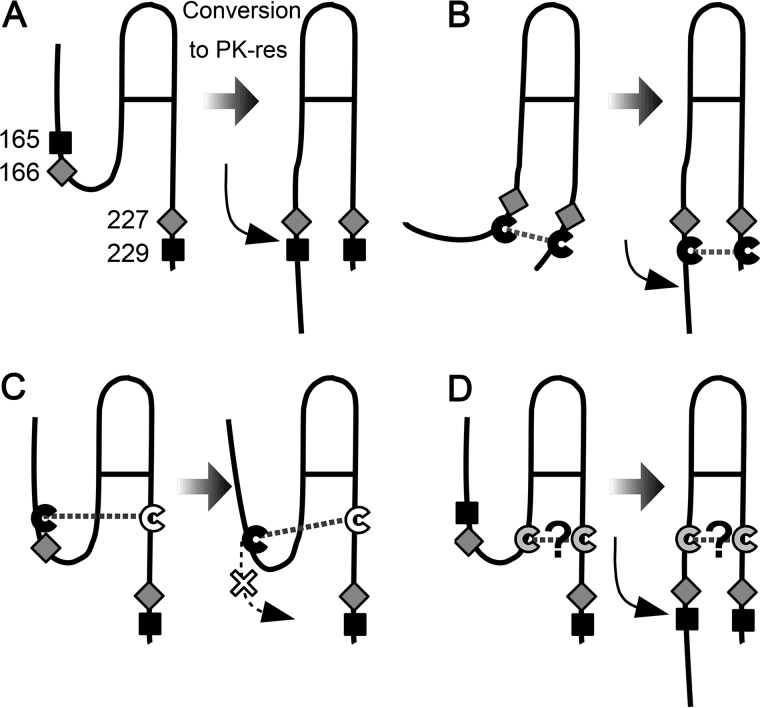
**A model to explain the discrepancy between high PrP expression and efficient PK-res conversion of convertible mutants.** *A,* hypothetical positional changes of H1∼H2 in the PrP^Sc^-dependent conversion reaction. *B,* a crosslink between residues 165 (or 166) and the distal portion of Ctrm deforms the conformation of PrP^C^, but does not severely affect the conversion because the position of H1∼H2 is suitable for conversion. *C,* a crosslink between residues 165 (or 166) and the proximal portion of Ctrm, *e.g.* 220C, might inhibit PK-res conversion by hampering the positional changes of H1∼H2. *D,* could there be a disulfide-crosslink connecting the more C-terminal H1∼H2 and the more proximal Ctrm, which would not hamper the conversion into PK-res?

**Figure 6.**
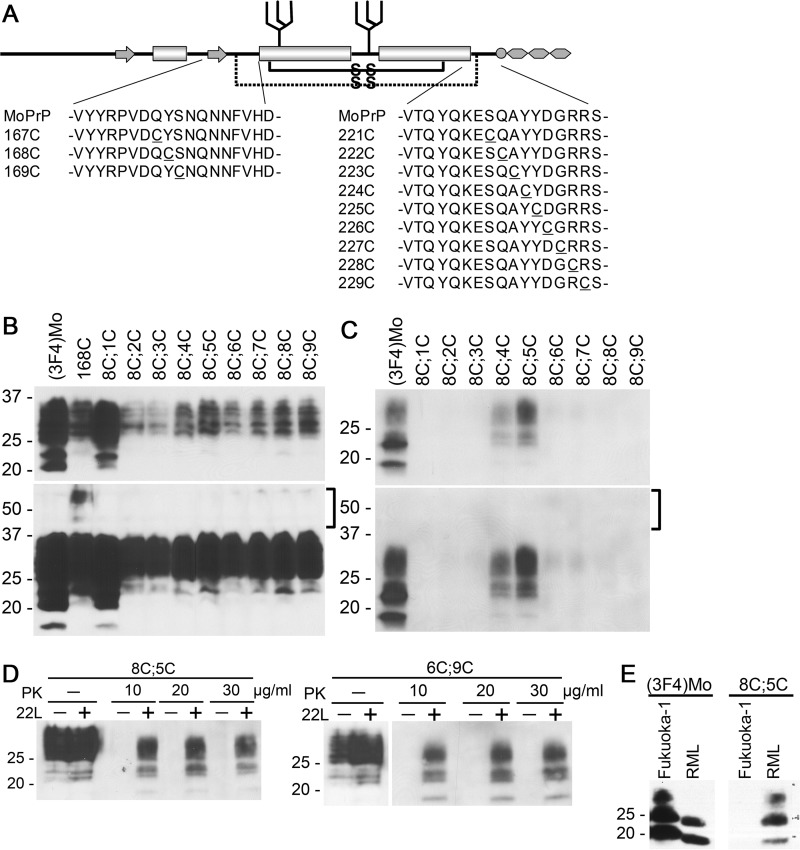
**Convertible mutants of the 168C;C-series support the hypothetical positional change of H1∼H2 during conversion to PK-res.** The *upper* and *lower panels* of blots represent images of short- and long-exposure to the same PVDF membranes, respectively. *A,* a schematic illustrating positions of substituted Cys residues of 167C;C-, 168C;C-, and 169C;C-series. *B,* PrP^C^ forms of 168C;C-series show similar banding patterns as WT PrP. Immunoblots with 3F4 mAb showing expression levels and PrP banding patterns of 168C;C-series. *Square brackets* denote positions of dimeric forms of mutant PrPs. *C,* conversion capacities of 168C;C-series. Immunoblots with 3F4 mAb demonstrating the levels of PK-res formation of the 168C;C-series. The *square brackets* indicate the position of the dimeric form. Like PrP^C^ forms, PK-res of the 168C;C-series also lack the dimeric forms. *D,* PK-res PrP formation of 8C;5C is PrP^Sc^ dependent, such as that of 6C;9C PrP. Immunoblots probed with 3F4 mAb comparing samples prepared from 22L-ScN2a and noninfected N2a cells, transfected with 8C;5C or 6C;9C, and digested with different concentrations of PK. *Left* and *right panels* show samples from cells transfected with 8C;5C and 6C;9C, respectively. Note that PK-res PrP of 8C;5C is only found in 22L-ScN2a cells, similar to 6C;9C. PK-digested and nondigested samples were on the same membrane and unnecessary lanes were removed. *E,* conversion of 8C;5C into the PK-res isoform is prion strain-dependent. Immunoblots were probed with 3F4 mAb comparing PK-res of (3F4)MoPrP and 8C;5C from transiently transfected Fukuoka1-infected or RML-infected N2a58 cells. Note that 8C;5C does not convert to PK-res PrP in Fukuoka1-infected cells while it converts in RML-infected cells.

The dependence of PK-res conversion of C;C-PrPs on *bona fide* PrP^Sc^ indicated that they are results of refolding reaction induced by PrP^Sc^. Tolerance of PK-res conversion reaction to specific disulfide-crosslinks, namely those of 6C;5C to 6C;9C, 5C;8C and 5C;9C, seemed to be reasonably explained with a model where H1∼H2 undergoes a positional change toward Ctrm during refolding into PK-res (Fig. 5*A*). This suggest that a disulfide-crosslink between Cys at positions 165 or 166 and Cys-229 does not interfere with the refolding process (Fig. 5*B*). This model predicted the existence of another disulfide-crosslink that might not interfere with the refolding reaction, bonding a more distal H1∼H2 residue and a more proximal Ctrm residue (Fig. 5, *C versus D*). To test this hypothesis, we created 168C;C-PrPs (Fig. 6*A*) and assessed their conversion efficiencies in 22L-ScN2a cells. Expression levels of 168C;C-PrPs in N2a cells were similar to that of 165C;C- and 166C;C-series mutants (Fig. 6*B*): 8C;1C showed expression comparable with (3F4)MoPrP, and 8C;4C and 8C;5C showed a slightly higher expression. In 22L-ScN2a cells, only 8C;4C and 8C;5C converted into PK-res forms at detectable levels (Fig. 6*C*) in a PrP^Sc^-dependent manner (Fig. 6*D*). When we combined 167C or 169C with Cys-scanning in Ctrm from 224 to 229 and 221 to 226, respectively, there were no detectable levels of PK-res.

### Prion strain dependence of PK-res conversion of 8C;5C

We previously hypothesized that a PrP molecule has multiple PrP^C^–PrP^Sc^ interfaces including H1∼H2, and that usage of interfaces varies among different prion strains (33). To test this hypothesis, we expressed 8C;5C in Fukuoka1- or RML-infected N2a58 cells and monitored its conversion into PK-res. Surprisingly, PK-res of 8C;5C were seen only in RML-infected cells, whereas it was completely absent in Fukuoka1-infected cells (Fig. 6*E*).

## Discussion

Our studies with C;C-PrP molecules provide new insights into the biology of cellular prion conversion, in particular into regional structures of PrP^Sc^ in prion conversion, potential prion strain differences, and anti-prion effects of the Q218K substitution. Following are detailed discussions.

### Conformations of PrP^C^ isoforms of C;C-PrPs and consequences in cultured cells

A variation in expression levels was seen among C;C-PrPs, with some comparable with that of (3F4)MoPrP and others showing lower expression. What could explain this? As mentioned above, a disulfide-crosslink of human PrP between residues 166 and 221 or 225 (human numbering) maintained or even stabilized the global structure of PrP^C^ in the native conformation (25) (26). Likewise, the corresponding residues of mouse PrP^C^, residues 165 and 220, are close enough to form a stable disulfide bond (*c.f*. PDB code: 2L39 (14)), and the disulfide-crosslink should not interfere with the native PrP^C^ conformation. The predicted conformation possibly explains the high expression level of 5C;0C. The native conformation is thermodynamically stable with the least molecular surface hydrophobic patches, which are targeted by ER or post-ER quality control systems, hence least elimination by those systems. To the contrary, the low-expression C;C-PrPs might have aberrant conformations and be actively eliminated by the quality control systems. Although the replaced residues of low-expression C;C-PrPs are theoretically too far apart to form a disulfide-crosslink, possibly structural fluctuations of H1∼H2 and Ctrm let them crosslink and fixate PrP^C^ of C;C-PrP at an aberrant conformation.

### Implications on regional structures of PrP^Sc^

The 165C;C-, 166C;C-, and 168C;C-series showed distinct patterns of convertible PrP mutants. This demonstrates that positioning of the disulfide-crosslink between H1∼H2 and Ctrm is a critical determinant of convertibility rather than positions of Cys itself. Because a disulfide-crosslink between two regions fixates their relative positioning and regional structures (34), the successful introduction of artificial disulfide-crosslinks to a protein, without affecting the global conformation, is highly informative about regional structures of the protein. This approach was used in investigation of regional structures of PrP^C^ and PrP fibrils as well (13, 25, 26). The convertible C;C-PrPs are informative about the regional structures of PrP^Sc^, or PK-resistant intermediates in the conversion pathway, because it suggests that their fixated assignment of H1∼H2 and Ctrm does not have to greatly change in the conversion reaction. Moreover, the striking discrepancy between most expressed and most converted PrPs of 165C;C-, 166C;C-, and 168C;C-series (namely 5C;0C *versus* 5C;9C, 6C;1C *versus* 6C;9C, and 8C;1C *versus* 8C;5C, respectively) demonstrates that the positioning of H1∼H2 and Ctrm suitable for achieving the native PrP^C^ conformation and for PrP^Sc^ conversion are distinct. Collectively, these results imply a substantial positional change of the H1∼H2 region, which is normally stabilized onto H2 and H3 by multiple hydrogen bonds and salt bridges, toward Ctrm in the prion conversion reaction in 22L-ScN2a cells, as illustrated in Fig. 6*D*. This point of view also would help explain why destabilization of the native PrP^C^ conformation leads the way for conversion of PrP into PrP^Sc^.

Among the convertible C;C-PrP constructs the 8C;4C and 8C;5C mutants are of particular interest. Kurt *et al*. (35) have reported that replacement of tyrosine at residue 168 with aromatic residues does not affect conversion of the mutant PrPs in *in vitro* conversion reactions. Mutant PrPs with Y168F or Y224F substitution also normally convert in RML-infected N2a cells (36). Possibly, aromatic–aromatic interactions between Tyr-168 and Tyr-224 or Tyr-225 contribute to the conversion of WT PrP by bonding H1∼H2 to Ctrm. The prion strain dependence of PrP^Sc^ conversion of 8C;5C is probably the most important finding of this study. It demonstrates a strain–dependent importance of H1∼H2 and Ctrm in PrP^Sc^ formation, which strongly supports our hypothesis that a varied usage of interfaces underlies the strain diversity of PrP^Sc^ (33). Presumably, Fukuoka1 and RML PrP^Sc^ have differential structures in those regions. This finding is also consistent with the strain–specific resistance of mice expressing PrP with N170S (37), implying a strain–dependent impact of regional structures in H1∼H2. Besides, differences between ME7, 22L, and RML prion strains in the regional structures of PrP^Sc^ in the Ctrm region are also implied by their distinct immunoreactivity (38). Our proposed model specifically exemplifies regional structures that could explain those observations. The nonconvertible 8C;1C-PrP mutant is also interesting because an aromatic phenylalanine at residue 225 of deer PrP (corresponding to 221 of mouse PrP) renders resistance to CWD and SSBP/1 infection (39). If Tyr-172 (corresponding to 168 of mouse PrP) and Phe-225 of deer PrP^C^ are close enough for aromatic–aromatic interactions, they can stabilize the PrP^C^ conformation and eventually negatively affect prion conversion.

Another possible explanation for the strain–dependent convertibility of C;C-PrPs implicates regional structures of the monomeric isoform of C;C-PrPs. It is conceivable that stabilization of the native conformation would protect against conversion into PrP^Sc^ and, by contrast, its destabilization precipitates conversion. If PrP^Sc^ molecules of different prion strains have distinct amyloid core regions that initiate the conversion process of the whole molecule, efficiency of the regional conversion could greatly affect the conversion efficiency of the entire molecule. The strain–dependent convertibility of C;C-PrPs could be interpreted from this viewpoint: the crosslinks of the convertible C;C-PrPs destabilize certain regions of PrP, which are critical for 22L and RML strains and enhance conversion by those strains, whereas the same region is not important for the Fukuoka1 strain. Indeed, strain diversity of Sup35 yeast prions was reported to be attributable to this type of mechanism (40).

Currently, in-register parallel β-sheet models (8) and four-rung β-solenoid models (41) are discussed as structural models for PrP^Sc^. The focus of the present study is on the regional structure and entire structures of PrP^Sc^ are out of scope. However, some implications on the structure of PrP^Sc^ can also be drawn from our data. They indicate that H1∼H2 and the region C-terminal to Cys-213 are situated side-by-side over a stretch, presumably interacting through residue side chains. Considering that the current four-rung solenoid model of PrP^Sc^ does not seem to postulate such anti-parallel arrangement of β-sheets, the present results are more compatible with the in-register parallel β-sheet model. The insights obtained by the present study hopefully contribute to refinement of parallel β-sheet models of PrP^Sc^.

Important aspects in the prion conversion process of the described C;C-PrP mutants that have to be addressed by further experimental investigation include whether the convertible mutants can convert in the absence of co-existing WT PrP and whether they also inherit *bona fide* prion infectivity. This could be proven by expressing convertible C;C-PrPs in cells without endogenous PrP, infecting them *de novo* with mouse-adapted scrapie and then analyzing PrP^Sc^ propagation and infectivity of the cells, *e.g.* in mouse bioassays. If they can mediate infectivity in mouse models that may even develop distinct clinicopathological pictures, C;C-PrPs might provide important new insights into structure–phenotype relationships of PrP^Sc^. In this regard, transgenic mice expressing C;C-PrPs might be very informative.

### Effect of Q218K substitution on conversion efficiency of 6C;9C

The Lys-219 polymorphism of human PrP is protective against sporadic CJD, but the exact underlying mechanism is yet to be determined. The partial suppression of the effects of Q218K by the disulfide-crosslink of 6C;9C implies an involvement of the positional relationship of H1∼H2 and Ctrm. Interestingly, although Lys-219 of human PrP slows or modifies pathologies of sporadic CJD (28, 29, 31, 42), new-variant CJD might not be affected or even be enhanced (43, 44). This is consistent with our view that the significance of the positional relationship of those regions is prion strain-dependent.

### Mechanism of diglycoform predominance of PrP^Sc^

Diglycoform predominance of PrP^Sc^ is characteristic of new variant CJD and some forms of familial CJD (12). It also occurs in experimental transmission to elk or bank vole (20, 45). The diglycoform predominance of PK-resistant PrP^Sc^ derived from C;C-PrPs in 22L-ScN2a cells implies that positional relationships between H1∼H2 and Ctrm of the nascent PrP^Sc^ is one determinant of the glycoform ratio. One possible mechanism is that the crosslink between H1∼H2 and Ctrm is advantageous for conversion of the di-glycoforms of C;C-PrPs. As the di-glycosylated forms of PrP^C^ are much more abundant than the other glycoforms, theoretically even a small improvement in conversion efficiency of the di-glycoform can change the glycoform ratio.

In conclusion, the described C;C-PrP mutants are unique and are promising experimental tools that can help to further elucidate the still mysterious biology of cellular prion conversion. In light of *prion-like* mechanisms described in a variety of major human neurodegenerative diseases, insights into the molecular mechanisms that let a normal protein convert into an abnormal isoform are of more general importance.

## Experimental procedures

### Reagents and antibodies

All media and buffers for cell culture and Lipofectamine LTX Plus were from Life Technologies Corp. Plasmid purification kit, DNA gel extraction kit, site-directed mutagenesis kit, detergents (including Triton X-100, deoxycholic acid, Triton X-114, Tween 20 and SDS), PK, anti-PrP monoclonal antibodies (mAb) 4H11 and 3F4 (recognizing residues 108–111 of human PrP), and all secondary antibodies were as previously reported (22). Iodoacetamide (IAA), dithiothreitol (DTT), Glu-C endopeptidase (V8 protease), and anti-FLAG polyclonal antibody were purchased from Sigma.

### Site-directed mutagenesis

All primers for site-directed mutagenesis were ordered from Integrated DNA Technologies, Inc. (Coralville, IA) and are listed in Table S1. Mutations were made with a QuikChange Site-directed Mutagenesis Kit (Agilent Technologies, Inc., Santa Clara, CA) according to the manufacturer's instructions. Sequences of mutant PrPs were determined by Eton Bioscience, Inc. (San Diego, CA).

### Cell culture, transient transfection, and analysis of PK-resistant fragments

Transient transfection of mouse neuroblastoma cell lines with or without persistent scrapie infection (22L-ScN2a or N2a, respectively), and procedures for preparation of samples of transfected N2a or 22L-ScN2a cells were as previously described (22), except for some modifications. Briefly, cells on 24-well plates were transfected with 0.3 μg/well of each plasmid with Lipofectamine LTX Plus (Life Technologies) for evaluation of expression or PrP^Sc^ levels of mutant PrPs. For evaluation of dominant-negative inhibition, 0.2 μg each of (3F4)MoPrP and mutant PrP were co-transfected. The Fukuoka1- and RML-infected N2a58 cells were also previously described (23, 24).

### SDS-PAGE and immunoblotting

The protocol for SDS-PAGE, development of blots, methods of densitometry, and quantification have been described previously (22). In the evaluation of expression or PrP^Sc^ levels of C;C-PrPs, we applied the whole sample prepared from a well of equally plated cells on 24-well plates per lane of the SDS-PAGE gel (22). This resulted in equal amounts of cells loaded per lane.

### Digestion with V8 protease

N2a cells, ∼60% confluent on 6-well culture plates, were transiently transfected with 1.0 μg/well of plasmid encoding the mutant PrP with Lipofectamine LTX. Next day, the medium was replaced with fresh medium and cells were cultured further at 37 °C in a CO_2_ incubator. 48 h after transfection, cells were rinsed once with phosphate-buffered saline (PBS) and then 1 ml/well of 1.5 mm IAA in PBS was overlaid and incubated for 10 min at 4 °C. After removal of IAA, cells were rinsed once with PBS without calcium and magnesium (Ca/Mg) and incubated in 700 μl/well of 3 mm EDTA in PBS without calcium and magnesium at 4 °C for 5 min. Then, the cells were mechanically detached by pipetting and collected in a tube. The cell suspension was centrifuged at 1,000 × *g* at 4 °C for 5 min and the supernatant was discarded. 400 μl of phosphate-buffered 2% Triton X-114 lysis buffer was added, cells were resuspended by vortexing for ∼10 s, and incubated on ice for 30 min, with a few seconds of vortexing from time to time. The lysate was then centrifuged at 16,100 × *g* at 4 °C for 1 min and the supernatant was transferred to a screw-cap tube as Triton X-114 lysate. PrP was concentrated by Triton X-114 extraction and methanol/chloroform precipitation as previously described (22). The pelleted proteins after methanol/chloroform precipitation were dissolved in 0.5% SDS in 50 mm sodium bicarbonate on a shaking incubator (Thermomixer; Eppendorf AG, Germany), at 95 °C for 10 min with shaking at 1,400 rpm. After the pellet was completely dissolved, the solution was diluted with 4-fold volume of 200 mm sodium bicarbonate to dilute SDS concentration, so that V8-protease efficiently digests PrP. After addition of 2 μl of V8-protease (2.5 units/μl), the solution was incubated at 37 °C for 1 h. Finally, one-fourth volume of 5× sample buffer with or without DTT was added and boiled. For re-probing of the PVDF membrane, the membrane was incubated in 100% methanol for 20 min, washed in Tris-buffered saline-Tween, and incubated with another primary antibody in 5% milk in Tris-buffered saline-Tween.

### Immunofluorescence analysis

3F4 expression related to CtxB labeling. Medium of N2a cells transfected with various C;C-PrP constructs for 72 h was replaced with ice-cold extracellular solution (ECS) for 5 min for pre-chilling prior to CtxB live cell labeling. Then cells were incubated with 2 μg/ml of 488-CtxB (Thermo Fisher; C34775) in cold ECS for 30 min at 4 °C. After washing 3 times in cold ECS, cells were fixed in 4% paraformaldehyde for 30 min at room temperature. Fixed cells were then permeabilized in PBS containing 5% FBS and 0.5% Triton for 30 min. Primary antibody 3F4 (Biolegend; 800307) was used at 1:1000 dilution for overnight incubation at 4 °C, and Alexa Fluor 555 goat anti-mouse secondary antibody (Thermo Fisher) was incubated for 30 min at room temperature. All images were taken under the same acquisition settings from a Zeiss 700 confocal microscope, and fluorescence was sequentially collected under standard 488 and Cy3 filters. 488-CtxB–labeled cell-surface lipid raft signal was used to define the “surface” contour, the same region of interest was copied to the Cy3 channel for quantifying surface 3F4 fluorescence intensity, and the whole cell fluorescence was measured from the same cell outline from Cy3 channel. For epifluorescence analysis, procedures for transient transfection of cells, fixation, permeabilization, and immunolabeling were as reported previously (22), except that samples were analyzed on an epifluorescence microscope, Olympus IX51, with objective lens Olympus LUCPlanFL N ×40 (0.60), and images were acquired with Olympus DP2-BSW software.

### Molecular dynamic simulation

The detailed protocol is provided under the supporting “Materials and Methods”.

## Author contributions

Y. T., N. N., and H. M. S. conceptualization; Y. T. and H. M. S. resources; Y. T., N. N., and H. M. S. data curation; Y. T., L. L., C. M.-W., H. O., and N. N. formal analysis; Y. T. and H. M. S. supervision; Y. T., N. N., and H. M. S. funding acquisition; Y. T. and N. N. validation; Y. T., L. L., C. M.-W., H. O., and N. N. investigation; Y. T., L. L., C. M.-W., H. O., and N. N. methodology; Y. T., L. L., C. M.-W., N. N., and H. M. S. writing-original draft; Y. T., N. N., and H. M. S. project administration; Y. T., N. N., and H. M. S. writing-review and editing.

## Supplementary Material

Supporting Information

## Footnotes

3 The abbreviations used are:

PrP^Sc^: abnormal isoform of PrP
PrP: prion protein
PrP^C^: normal isoform of PrP
C;C-PrP: PrP with two cysteine substitutions
PK-res: proteinase K-resistant isoform of PrP
H1∼H2: the region between the first and the second helix
Ctrm: the C-terminal region of H3
PK: proteinase K
ER: endoplasmic reticulum
PVDF: polyvinylidene difluoride
CJD: Creutzfeldt-Jakob disease
CWD: chronic wasting disease
CtxB: cholera toxin B
IAA: iodoacetamide
ECS: extracellular solution
PDB: Protein Data Bank.
