# Supplementary material for: Disulfide-crosslink scanning reveals prion–induced conformational changes and prion strain–specific structures of the pathological prion protein PrPSc
Source: J Biol Chem. 2018 Jun 22;293(33):12730–40. doi: 10.1074/jbc.RA117.001633 (PMC6102138; doi:10.1074/jbc.RA117.001633)
Supplement: Supporting Information [file supp_293_33_12730__index.html]

Disulfide-crosslink scanning reveals prion–induced conformational changes and prionstrain–specific structures of the pathological prion protein PrPSc — Disulfide-crosslinked PrP can be converted by PrPSc — Disulfide-crosslink scanning reveals prion–induced conformational changes and prion strain–specific structures of the pathological prion protein PrPSc — Disulfide-crosslinked PrP can be converted by PrPSc — Supporting Information 

# Disulfide-crosslink scanning reveals prion–induced conformational changes and prion strain–specific structures of the pathological prion protein PrPSc

## Supporting Information

- Supporting Information - Supporting Information
