# Supplementary material for: Disulfide-crosslink scanning reveals prion–induced conformational changes and prion strain–specific structures of the pathological prion protein PrPSc
Source: J Biol Chem. 2018 Jun 22;293(33):12730–40. doi: 10.1074/jbc.RA117.001633 (PMC6102138; doi:10.1074/jbc.RA117.001633)
Supplement: Supporting Information [file supp_RA117.001633_134909_2_supp_154249_pbbnb5.pdf]

Disulfide-crosslink scanning reveals prion–induced conformational changes and prion strain–specific structures of the pathological prion protein PrP^{Sc}

Yuzuru Taguchi, Li Lu, Cristobal Marrero-Winkens, Hiroki Otaki,
Noriyuki Nishida and Hermann M. Schatzl

Supplementary Figure 1

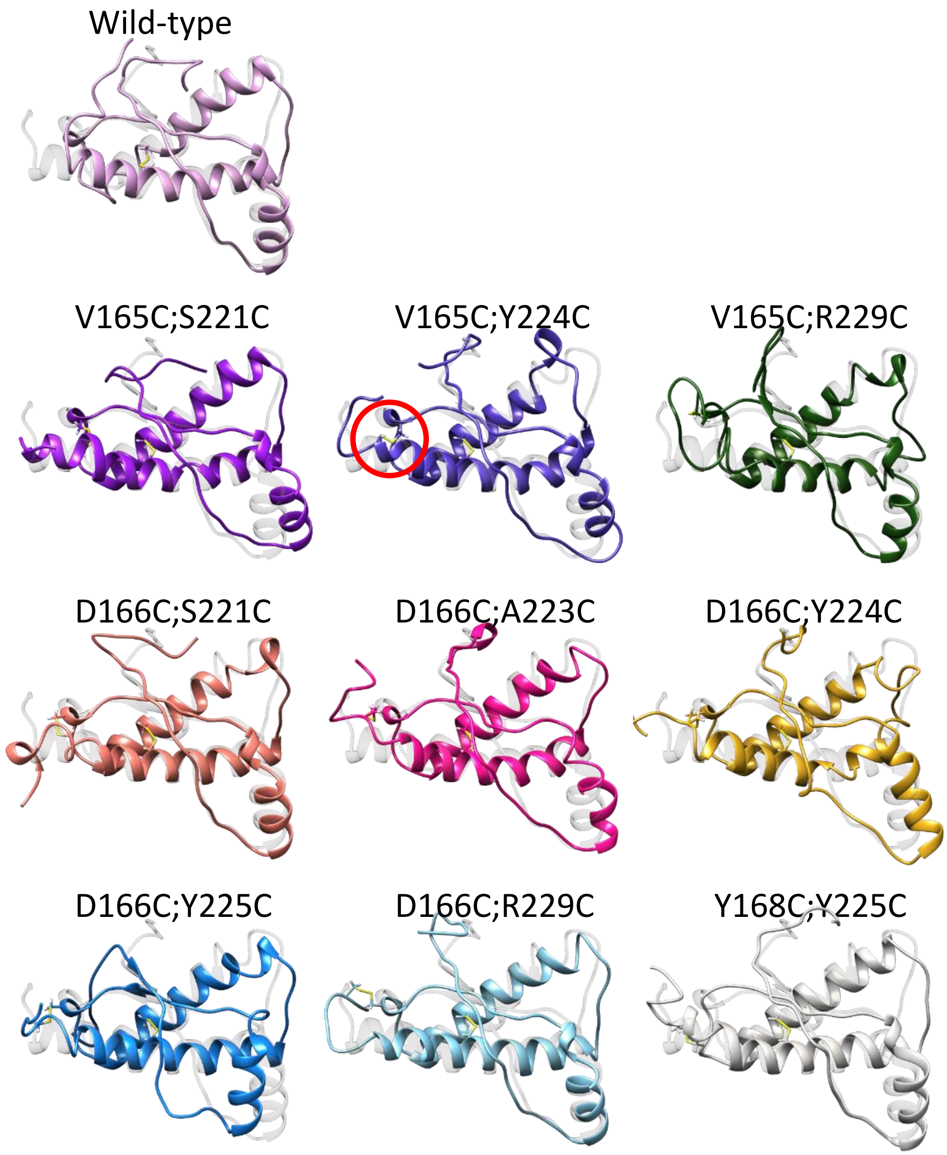

FIGURE S1. Images of representative C;C-PrP mutants after 50 ns of molecular dynamic simulation. The background gray images behind each image represent the template native conformation of the mouse PrP (PDB ID: 2L39, model 1). Note that each C;C-PrP has two disulfide bonds (yellow bonds), the native one between H2 and H3, and the newly-introduced one between the loop region and the C-terminal region, e.g. within the red circle in "V165C;Y224C". Interestingly, the conformations of C;C-PrPs were not severely distorted despite the artificial disulfide crosslinks, suggesting structural robustness of the globular domain of PrP.

Supplementary Figure S2

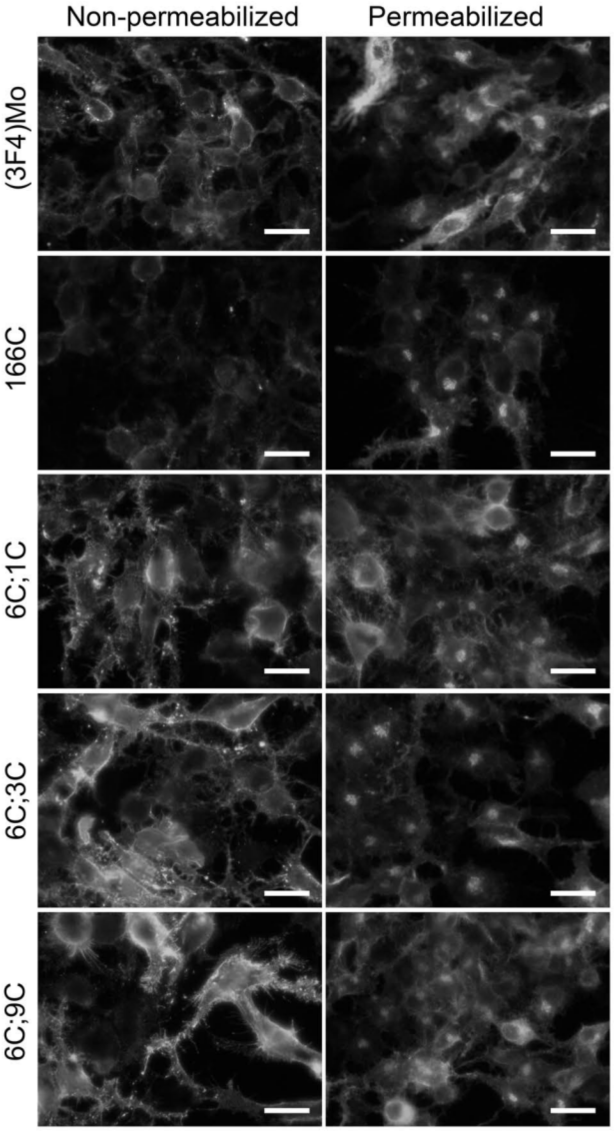

FIGURE S2. 166C;C-series mutants show the same subcellular localization as wild-type mouse PrP. Epifluorescence microscopy images using 3F4 mAb, with or without permeabilization. All of the C;C-constructs exhibit the same localization patterns as wild-type, i.e. at the cell-surface and in the perinuclear regions. Scale bars, 25 μm.

Supplementary Protocol

Material & Methods

Modeling Structure

In addition to WT, we carried out MD simulation of mutants 165C;221C, 165C;224C and 165C;229C from the 165C;C-series, 166C;221C, 166C;223C, 166C;224C, 166C;225C, and 166C;229C from the 166C;C-series, and 168C;225C from the 168C;C-series. The mutants were modeled with I-TASSER online server [1][2][3], where structure of WT PrP protein (2L39, model 1) was used for template. In order to minimize the electrostatic interactions at the N- and C-termini, we modified the termini with acetylation and *N*-methylation, respectively, using PyMOL. Modeled protein was solvated with a rhombic dodecahedron water box with minimum wall distance of 12 Å. Na^+ and Cl^- ions were randomly placed to neutralize the system and yield a net concentration of 150 mM NaCl.

MD simulation

For all MD simulations, we used GROMACS software [4] with AMBER ff99SB-ILDN force fields [5] for the PrP proteins and the TIP3P water model [6]. The system was minimized for 4,000 steps with steepest descent method, followed by 2,000 steps with conjugate gradient method. During the minimization, heavy atoms of the proteins were restrained with a harmonic potential with the force constant of 10.0 kcal/mol•$Å^2$, which was gradually decreased to 2.5 kcal/mol•$Å^2$ to relax the disulfide bonds.

After the minimization, the temperature of the system was increased from 0 to 310 K during 1 ns simulation with the restraints. Next, 1 ns of equilibration run was performed by gradually reducing the restraints from 10.0 kcal/mol•$Å^2$ to zero and subsequent equilibration was performed in the NPT ensemble for 2 ns at 310 K and 1 bar. Production runs were carried out for 50 ns in the NPT ensemble at 310 K and 1 bar.

We used velocity-rescaling scheme [7] and Berendsen scheme [8] for thermostat and barostat, respectively. The LINCS algorithm [9] was used to constrain all bonds with hydrogen atoms, allowing the use of 2 fs time step. Electrostatic interactions were calculated with the Particle-mesh Ewald method [10]. The cutoff length was set to 12 Å for Coulomb and van der Waals interactions. The Verlet cutoff scheme [11] was used for neighbor searching. For each production run, trajectory snapshots were saved at every 10 ps.

References

1. Yang J, Yan R, Roy A, Xu D, Poisson J, Zhang Y. The I-TASSER suite: Protein structure and function prediction. Nat Methods. Nature Publishing Group; 2014;12: 7–8. doi:10.1038/nmeth.3213
2. Roy A, Kucukural A, Zhang Y. I-TASSER: a unified platform for automated protein structure and function prediction. Nat Protoc. Nature Publishing Group; 2010;5: 725. Available: http://dx.doi.org/10.1038/nprot.2010.5
3. Zhang Y. I-TASSER server for protein 3D structure prediction. BMC Bioinformatics. 2008;9:

1–8. doi:10.1186/1471-2105-9-40

4. Abraham MJ, Murtola T, Schulz R, Páll S, Smith JC, Hess B, et al. GROMACS: High performance molecular simulations through multi-level parallelism from laptops to supercomputers. SoftwareX. 2015;1–2: 19–25. doi:https://doi.org/10.1016/j.softx.2015.06.001
5. Lindorff-Larsen K, Piana S, Palmo K, Maragakis P, Klepeis JL, Dror RO, et al. Improved side-chain torsion potentials for the Amber ff99SB protein force field. Proteins Struct Funct Bioinforma. Wiley Subscription Services, Inc., A Wiley Company; 2010;78: 1950–1958. doi:10.1002/prot.22711
6. Jorgensen WL, Chandrasekhar J, Madura JD, Impey RW, Klein ML. Comparison of simple potential functions for simulating liquid water. J Chem Phys. American Institute of Physics; 1983;79: 926–935. doi:10.1063/1.445869
7. Bussi G, Donadio D, Parrinello M. Canonical sampling through velocity rescaling. J Chem Phys. American Institute of Physics; 2007;126: 14101. doi:10.1063/1.2408420
8. Berendsen HJC, Postma JPM, van Gunsteren WF, DiNola A, Haak JR. Molecular dynamics with coupling to an external bath. J Chem Phys. American Institute of Physics; 1984;81: 3684–3690. doi:10.1063/1.448118
9. Hess B. P-LINCS: A Parallel Linear Constraint Solver for Molecular Simulation. J Chem Theory Comput. American Chemical Society; 2008;4: 116–122. doi:10.1021/ct700200b
10. Essmann U, Perera L, Berkowitz ML, Darden T, Lee H, Pedersen LG. A smooth particle mesh Ewald method. J Chem Phys. American Institute of Physics; 1995;103: 8577–8593. doi:10.1063/1.470117
11. Páll S, Hess B. A flexible algorithm for calculating pair interactions on SIMD architectures. Comput Phys Commun. 2013;184: 2641–2650. doi:https://doi.org/10.1016/j.cpc.2013.06.003

Primers used for the site-directed mutagenesis

165C	5'	CTACAGGCCATGCGATCAGTACAGCAACCAGAACAACTTCGTGCACGAC
r-165C	5'	GTTGCTGTACTGATCGCATGGCCTGTAGTACAC
166C	5'	TACTACAGGCCAGTGTGCCAGTACAGCAACCAG
r-166C	5'	CTGGTTGCTGTACTGGCACACTGGCCTGTAGTA
167C	5'	GGC CAG TGG ATT GCT ACA GCA ACC AGA ACA ACT TCG TGC ACG
r-167C	5'	GTA GCA ATC CAC TGG CCT GTA GTA CAC TTG GTT AGG GTA GCG G
168C	5'	GTG GAT CAG TGC AGC AAC CAG AAC AAC TTC GTG CAC GAC TGC
r-168C	5'	GTT GCT GCA CTG ATC CAC TGG CCT GTA GTA CAC TTG GTT AGG
169C	5'	GTGGATCAGTACTGCAACCAGAACAACTTCGTGCACGACTGC
r-169C	5'	GTTGCAGTACTGATCCACTGGCCTGTAGTACACTTGGTTAGG
ComSen220-223	5'	CTATTACGACGGGAGAAGATCCAGCAGCACCGTGCTTTTC
r-220C	5'	CTTCTCCCGTCGTAATAGGCCTGGGAGCACTTCTGGTACTGGGTG
r-221C	5'	CTTCTCCCGTCGTAATAGGCCTGGCACTCCTTCTGGTACTGG
r-222C	5'	CTTCTCCCGTCGTAATAGGCGCAGGACTCCTTCTGGTACTGG
r-223C	5'	CTTCTCCCGTCGTAATAGCACTGGGACTCCTTCTGGTACTGG
224C	5'	GGAGTCCCAGGCCTGTTACGACGGGAGAAG
r-224C	5'	CTTCTCCCGTCGTAACAGGCCTGGGACTCC
225C	5'	GAGTCCCAGGCCTATTGCGACGGGAGAAGATCC
r-225C	5'	GGATCTTCTCCCGTCGCAATAGGCCTGGGACTC
226C	5'	CCCAGGCCTATTACTGCGGGAGAAGATCCAGC
r-226C	5'	GCTGGATCTTCTCCCGCAGTAATAGGCCTGGG
227C	5'	CAGGCCTATTACGACTGCAGAAGATCCAGCAGC
r-227C	5'	GCTGCTGGATCTTCTGCAGTCGTAATAGGCCTG
228C	5'	GCC TAT TAC GAC GGG TGC AGA TCC AGC AGC ACC
r-228C	5'	GCT GCT GGA TCT TCT GCA GTC GTA ATA GGC CTG
229C	5'	TAT TAC GAC GGG AGA TGC TCC AGC AGC ACC GTG
r-229C	5'	CAC GGT GCT GCT GGA GCA TCT CCC GTC GTA ATA
230C	5'	CGACGGGAGAAGATGCAGCAGCACCGTGC
r-230C	5'	GCACGGTGCTGCTGCATCTTCTCCCGTCG
231C	5'	CGGGAGAAGATCCTGCAGCACCGTGCTTTTC
r-231C	5'	GAAAAGCACGGTGCTGCAGGATCTTCTCCCG
C178A	5'	GACGCCGTCAATATCACCATCAAGCAGCACACGGTCACCACC
r-178A	5'	GGTGATATTGACGGCGTCGTGCACGAAGTTGTTCTGGTTGCTG
C213A	5'	GATGGCCGTCACCCAGTACCAGAAGGAGTCCCAGGCCTATTACG
r-213A	5'	CTGGGTGACGGCCATCTGCTCCACCACGCGCTCCATCATCTTC
218K	5'	GTGCGTCACCCAGTACAAGAAGGAGTCCCAGGCC
r-218K	5'	GGCCTGGGACTCCTTCTTGTACTGGGTGACGCAC
r-FLAG (for 0-4C)	5'	CGTCATCGTCTTTGTAGTCAGATCTTCTCCCGTCGTAATAGG
r-5C-FLAG	5'	CGTCATCGTCTTTGTAGTCAGATCTTCTCCCGTCGCAATAGG
r-7C-FLAG	5'	CGTCATCGTCTTTGTAGTCAGATCTTCTGCAGTCGTAATAGG
r-9C-FLAG	5'	CGTCATCGTCTTTGTAGTCAGAGCATCTCCCGTCGTAATAGG
231FLAG	5'	CTACAAAGACGATGACGATAAATCCAGCAGCACCGTGCTTTTC
